# Rapid and Complete Remission of Metastatic Adrenocortical Carcinoma Persisting 10 Years After Treatment With Mitotane Monotherapy: Case Report and Review of the Literature: Erratum

**DOI:** 10.1097/01.md.0000496598.29629.d2

**Published:** 2016-09-09

In the article “Rapid and Complete Remission of Metastatic Adrenocortical Carcinoma Persisting 10 Years After Treatment With Mitotane Monotherapy: Case Report and Review of the Literature”,^1^ which appeared in Volume 95, Issue 13 of *Medicine*, errors appeared in the affiliations footnote. The article has since been corrected online.
